# The effectiveness of the South African Triage Toll use in Mahalapye District Hospital – Emergency Department, Botswana

**DOI:** 10.4102/phcfm.v8i1.1030

**Published:** 2016-07-26

**Authors:** Stephane T. Tshitenge, Gboyega A. Ogunbanjo, Deogratias O. Mbuka

**Affiliations:** 1 Department of Family Medicine and Public Health, University of Botswana, Botswana; 2 Department of Family Medicine and Primary Health Care, Sefako Makgatho Health Sciences University, South Africa

## Abstract

**Background:**

The study aimed to determine the proportion of each priority level of patients, time of performance in each priority level, and the reliability of the South African Triage Scale (SATS) tool at the Mahalapye District Hospital - Emergency Department (MDH-ED), a setting where the majority of the nurses were not formally trained on the use of the SATS.

**Methods:**

This was a cross-sectional study using case records in MDH-ED from 1 January 2014 to 31 December 2014. A panel of experts from the Mahalapye site of the Family Medicine Department, University of Botswana, reviewed and scored each selected case record that was compared with the scores previously attributed to the nurse triage.

**Results:**

From the 315 case records, both the nurse triage and the panel of expert triage assigned the majority of cases in the routine category (green), 146 (46%) and 125 (40%), respectively, or in the urgent category (yellow), they assigned 140 (44%) and 111 (35%) cases, respectively.

Overall, there was an adequate agreement between the nurse triage and the panel of expert triage (*k* = 0.4, 95% confidence interval: 0.3–0.5), although the level of agreement was satisfactory.

**Conclusion:**

Findings of the study reported that the profile of the priority-level categories in MDH-ED was made in the majority of routine and urgent patients, only the routine and the emergency patients were seen within the targeted time and they had a satisfactory level of reliability (between 0.4 and 0.6).

## Introduction

With globalisation and lifestyle changes, entry points of health institutions in developing countries encounter challenges in managing the increased number of patients with communicable and non-communicable diseases. In 2010, to deal with their busy emergency centres, Botswana health institutions decided to use the South African Triage Scale (SATS) tool as there was no formal triage system in place prior to the use of the tool.^1^

The SATS tool consists of the Triage of Early Warning Score (TEWS) and a series of clinical discriminators.^2,3,4^The TEWS includes the patient mobility, presence of trauma and a physiological scoring system from the Modified Early Warning Score.^2,3,4,5^

SATS helps to sort patients into four priority levels, namely, emergency, very urgent, urgent and routine (not urgent), that are represented in colour codes with a target or ideal time that a patient can safely wait to be seen by the doctor.^2^

These priority levels correspond to red (should be seen immediately), orange (should be seen within 10 min), yellow (should be seen within 10 min and 60 min) and green (should be seen within 1 h and 4 h), respectively, upon arrival at a health facility.^2,6,7^

The SATS has been formally appraised in its implementation within and outside sub-Saharan Africa.^8,9,10,11,12,13^ It has a high reliability of up to 0.87 and helps to improve the waiting time of the categorised patients,^8,11,12,13^ as in the majority of cases these assessments are conducted after a formal and meticulous training of healthcare professionals who use the tool.

In 2012, the Mahalapye District Hospital (MDH) management assigned a task force team to review processes at the entry point of the hospital in order to solve the recurrent problem of patients overcrowding at the outpatient and emergency department (MDH-ED). It was then resolved that, from the entry point of MDH, which are the outpatient and MDH-ED, patients should be sorted and prioritised using the SATS before directing them to a specific unit of care. A team of two family medicine residents conducted a half-day training for nurses as they were previously trained and used the tool at Princess Marina Hospital, the tertiary referral for Botswana.

The majority of the trained nurses were later moved to other units or transferred out of the MDH, resulting in subsequent new nurses posted to MDH-ED who had to learn about the use of SATS from their colleagues or were self-trained.

Little is known about how the SATS tool performs in a limited-resource setting where healthcare professionals did not have formal training or were self-trained.

We hypothesised that, despite the self-training or the lack of a formal training of health professionals on the SATS tool, which will potentially reduce the accuracy in the categorisation of patients, the tool could still be useful in reducing waiting time and in prioritising patients in such setting.

The study aimed to assess the proportion of each priority level of patients, time of performance in each priority level and the reliability of the tool in MDH-ED.

## Methods

### Study design, setting and sampling

This was a cross-sectional study using case records in MDH-ED from 1 January 2014 to 31 December 2014.

The Mahalapye (Sub-District) is a rural sub-district located in the central part of Botswana about 200 km from Botswana’s capital city Gaborone. It has a population of 118 876 people.^13^ The MDH-ED attends to approximately 16 000 patients in a year, 80% of whom are self-referred. MDH-ED operates from 07:30 to 16:30 for the daytime shift and from 16:30 to 07:30 the next day for the night shift.

We conducted a pilot study to estimate the means ± standard deviation (s.d.) of the time spent by patients between the triage in at least two priority levels that could help in the sample size calculation. We used 48 case records randomly selected from the 2014 case records; the routine case records were 22 and the urgent case records were 26, the ratio was almost 1:1. The mean ± s.d. of the time spent by patients between the triage and the consultation of routine was 51 (74) min and for the urgent patients, it was 75 (85) min. We computed, from a study population of 16 000, a sample size of 311 case records as a minimum required with a study power of 0.80, a type I error rate of 0.05 and an additional 15% of the calculated sample size as the sample was not normally distributed.^14,15,16^

We used a systematic sampling method and selected every 51 case record (16 000 ÷ 311) from a random starting point determined by a table of random numbers. When the case record was unreadable or incomplete, the next case record was selected.

We excluded case records from patients who did not attend MDH-ED during the study period from 1 January to 31 December 2014.

### Data collection and analysis

The variables consisted of patients’ age, sex, accurate priority level as assigned by the nurse triage and the panel of experts, waiting time from triage to a consultation with a doctor or a consulting nurse. We summarised the data using the mean ± s.d. for normally distributed variables, the median ± interquartile range for skewed and the frequency in percentages for binomial. The Kruskal–Wallis rank test was used to compare the medians of the waiting time of consultation after the nurse triage. To find out whether there was a difference in medians of routine and urgent patients waiting time for a consultation after a nurse triage, we used the Mann–Whitney test.

A panel of experts (a family physician and a family medicine resident), from the Mahalapye site of the Family Medicine Department, University of Botswana with in-depth knowledge of the SATS and experience in its use, reviewed and scored each selected case record.

We used kappa to compare the nurse acuity level triage’s rating and the panel of expert’s rating. Reliability was described using the kappa statistic, where *κ* = 0 indicates an absence of agreement random result and *κ* = 1 shows total agreement between the two measurements. The agreement was classified as follows: poor (*κ* < 0.2), adequate (0.2 < *κ* < 0.4), satisfactory (0.4 < *κ* < 0.6), good (0.6 < *κ* < 0.8) and very good (0.8 < *κ* < 1).^17^

R software version 3.0.0 with R commander package version 1.9-6 was used to capture and analyse the data. The level of statistical significance was *p* = 0.05.

### Ethical considerations

The Health Research Unit of the Ministry of Health, Botswana, granted ethics approval for the study [PPME-13/8/1 PS V (342)]. The Mahalapye District Health Team Ethics Committee also approved the study and provided a waiver of consent, because the study required routinely maintained medical records [MH/DHMT/1/7/7 (5)]. To ensure confidentiality, no patient identifiers were used.

## Results

Three hundred and twenty-three case records were included in the study. Eight case records were discarded as they were incomplete, or unreadable, resulting in the analysed sample size of 315 records.

### Demographic characteristics of the study population

Of 315 case records, 170 [54%, 95% confidence interval (CI): 48%–60%] were female patients, and 145 (46%, 95% CI: 40%–52%) were male patients. The mean ± s.d. for age of case records was 30 ± 23 years. Daytime and call time/holidays shared almost the same number of cases [148 (47%) vs. 167 (53%), *p* = 0.5].

### Priority levels and clinical discriminators profile

**FIGURE 1:**
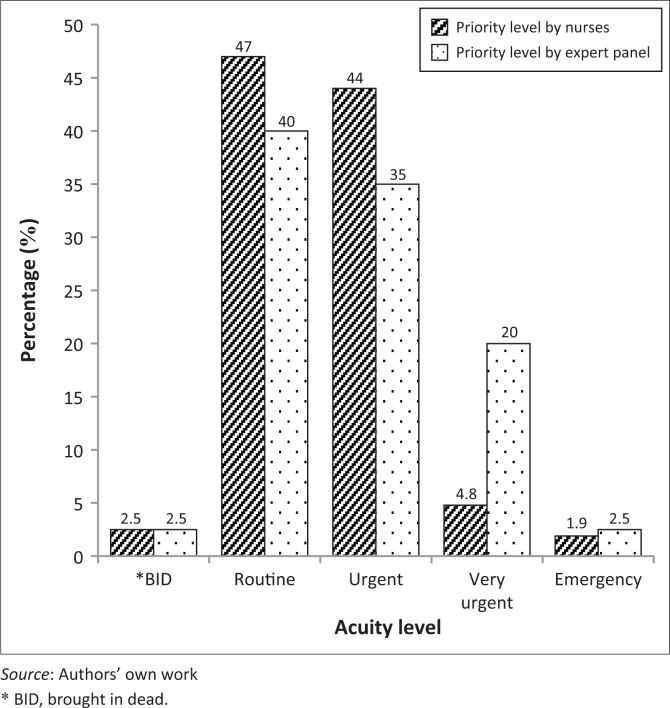
The proportion of each acuity level as triage nurses and research experts observed in Mahalapye District Hospital – Emergency Department in 2014.

Figure 1 and Table 1 summarise the proportion of cases per priority level as observed by the nurse triage and the panel of expert triage. Both nurse and panel of expert triages assigned the majority of cases in the routine category (green), 146 (46%) and 125 (40%), respectively; or in the urgent category (yellow), they assigned 140 (44%) and 111 (35%) cases, respectively. The panel of expert triage allocated about four times more cases in very urgent (orange) than the nurse triage [63 (20%) vs. 15 (2.8%), *p* < 0.001), whilst both triages allocated few patients in the emergency category (red)- 6 (1.9%) and 8 (2.5%) respectively.

**TABLE 1:** Frequency of case records per acuity level as observed in nurse triage and expert panel triage in Mahalapye District Hospital – Emergency Department in 2014.

Nurse triage	Panel of expert triage					Total (nurse triage)	
	
	BID	Routine	Urgent	Very urgent	Emergency	*n*	% (95% CI)
BID	8‡	0	0	0	0	8	2.5 (1.2–5.1)
Routine	0	103‡	32§	11§	0	146	46 (41–52)
Urgent	0	20†	76‡	41§	3§	140	44 (39–50)
Very urgent	0	1†	3†	10‡	1§	15	4.8 (2.8–7.9)
Emergency	0	1†	0	1†	4‡	6	1.9 (0.8–4.3)
**Total (*n*)**	**8**	**125**	**111**	**63**	**8**	**315**	**-**
**(Panel of expert triage) % (95% CI)**	**2.5 (1.2–5.1)**	**40 (34–45)**	**35 (30–41)**	**20 (16–25)**	**2.5 (1.2–5.1)**	**-**	**100%**

*Source*: Authors’ own work

BID, brought in dead; CI, confidence interval.

† Under triage;

‡ Agreement;

§ Over triage.

### Waiting time from triage until the patient was seen by a doctor or a consulting nurse

**FIGURE 2:**
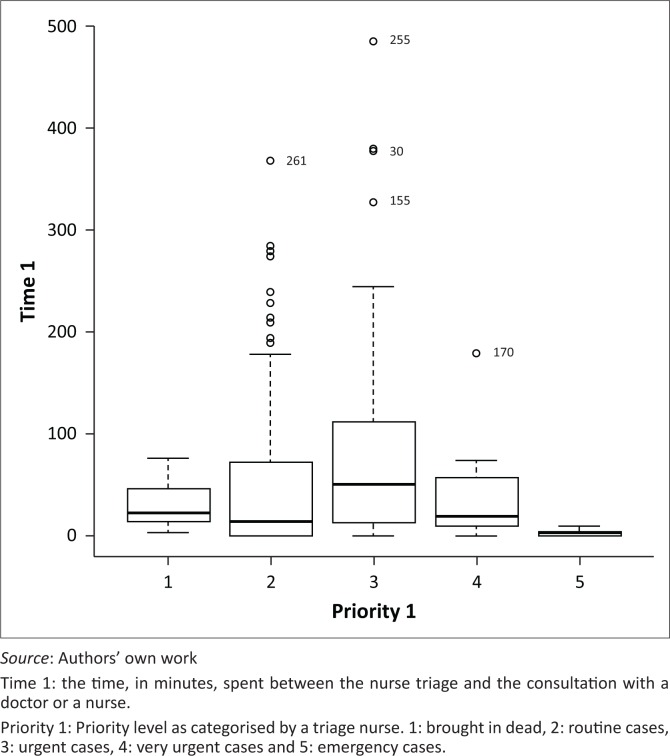
Boxplots of time, in minutes, spent between the nurse triage and the consultation with a doctor or a nurse by acuity level as categorised by the triage nurse in the Mahalapye District Hospital, in 2014.

Figure 2 illustrates waiting time from triage until the patient was seen by a doctor or a consulting nurse. From nurse triage until the patient was seen by a doctor or a consulting nurse, the median waiting time was 15 (0–74) min for routine cases, 51 (12–110) min for urgent case, 23 (10–69) min for a very urgent case and 3 (2–10) min for an emergency case.

Almost all routine patients were seen by a doctor or a consulting nurse within the target time of 4 h, and three-quarters (75%) of emergency patients were attended by a doctor within 10 min from the triage. Only close to half (50%) of the urgent patients and 50% of very urgent patients were attended to within the respective target of 60 min and 10 min.

There was a difference in priority levels’ medians of waiting time from the nurse triage until the patient was seen by a doctor or by a consulting nurse (*p* = 0.0004). Patients triaged in routine category spent less time to wait for a consultation by a doctor or a consulting nurse compared to those triaged in urgent category (15 min vs. 51 min, *p* = 0.001). In approximately one-quarter (25%) of patients in routine category, the nurse who triaged the patient was the same person who conducted the consultation.

### Agreement between the nurse triage and the panel of expert triage

Overall, there was adequate agreement between the nurse triage and the panel of expert triage (*k* = 0.4, 95% CI: 0.3–0.5). In two-thirds of cases [201, 64% (95% CI: 59%–69%)], the panel of experts allocation in various categories matched those of the nurses.

Between the nurse triage and the panel of expert triage, the level of agreement was satisfactory for the routine category (*k* = 0.6, 95% CI: 0.5–0.7), adequate for the urgent category (*k* = 0.4, 95% CI: 0.3–0.5), satisfactory for the emergency category (*k* = 0.6, 95% CI: 0.3–0.9) and poor for the very urgent category (*k* = 0.2, 95% CI: 0.1–0.3).

When there was disagreement between the nurse triage and the panel of expert triage, the panel of experts viewed that 1 in 10 (*n* = 26, 8.6%, 95% CI: 5.8%–12%) cases were undertriaged by nurses (Table 1). Cases at the very urgent category were the most undertriage (4.8% vs. 20%, *p* < 0.001). Whilst about one-third [88 (28%, 95% CI: 23%–33%)] were viewed by the panel of experts as an overtriage by nurses.

## Discussion

The MDH-ED profile was predominantly carried out with routine cases (40%), with few emergency cases (2.5%). The findings were in discordance with the findings from a South African study conducted in an ED primary level 1 of care that found that the quasi-majority of their patients (88%) were classified under ‘very urgent’ category.^11^ Another study conducted in a rural secondary hospital reported that when using SATS, all priority levels shared almost the same proportion of about one-third for routine category, another third for urgent category and the last third for both very urgent and emergency category.^5^ A study conducted at the emergency centre of Princess Marina Hospital in Gaborone, Botswana, found that the majority of patients who attended the ED were classified under the urgent category (39%) or very urgent category (36%).^9^ The findings from our study could be explained by the fact that MDH is a multilevel health facility (primary and secondary care) and majority of patients seen at this facility were self-referred patients.

In our study, almost all routine patients were seen for a consultation by a consulting nurse or a doctor within 4 h and three-quarters of emergency patients were seen for a consultation by a consulting nurse within 10 min. In approximately one-quarter of patients in the routine category, the nurse who triaged the patient was at the same time, the consulting nurse; this probably explained the time performance of the routine category. Patients who were classified as urgent or very urgent waited for a long time in comparison with the target waiting time of these categories.

When compared with a study from an emergency department in Bloemfontein, South Africa, where only 8% of patients were seen within the target time of an acuity level,^11^ one could argue that the findings from our study are encouraging. The factors such as number of patients and ratio of healthcare providers:patients should also be taken into consideration; the ED attendance ratios per month in the two studies were almost 2:1 in favour of the South African ED.^11^

In terms of agreement of measurement, when the nurse triage was compared to the expert panel triage, the overall agreement in the prioritising of patients was adequate (*k* = 0.4) for urgent patients, satisfactory for routine patients (*k* = 0.6) and poor in very urgent patients (*k* = 0.2). Our findings demonstrated a low level of agreement compared to the one reported from a study from Pakistan (0.87) ^13^ and a Botswana study that used a limited number of vignettes (25) after a meticulous training of healthcare workers (0.87).^8^ However, it was difficult to compare the later Botswana findings to the one from our study as their sample size was small, data were collected immediately after a meticulous training and it was not in a ‘real-life’ practice.

Our study reported that the triage nurse overtriaged cases (28%) more than they undertriaged (8.6%). The very urgent category was the most undertriaged group (4.8% vs. 20%, *p* < 0.001). A South African study also reported the tendency of the SATS to overtriage (67.9%) compared to undertriage (0.3%),^5^ whereas another study from a public hospital in South Africa reported an almost equal percentage between overtriage (25%) and undertriage (24%).^10^ However, from a tertiary facility in Ghana, undertriage proportion was more than overtriage (94% vs. 5.7%).^12^ Nurses with less experience on the use of triage tool had a tendency to undertriage patients.^18^

In our study, we could not determine who contributed between the trained nurses and the self-trained nurses to the high proportion of undertriage cases. The American College Surgeon Committee on Trauma defines the acceptable levels of undertriage of 5% and the acceptable levels of overtriage of 25%–35%,^19^ whilst Twomey et al.^2^ resolved that, when using SATS, a range of 10% undertriage and 15% overtriage were acceptable. In accordance to these standards, one could argue that the proportion of cases that were undertriaged and overtriaged in our study could be acceptable.

Despite the fact that healthcare professionals at MDH-ED did not have formal training or were self-trained, the SATS tool was still useful because of the agreement between the nurse triage and the panel of expert triage and in its ability to allow majority of patients to be seen within the target waiting time. The usefulness and adequacy of the tool was substantiated by the accurate level of priority categorisation and the on-target medians waiting time of 3 min for the emergency category, 51 min for the urgent category and 15 min for the routine category.

The exception was the very urgent category (median = 23 min), where only half of the patients were attended within the target waiting time for a consultation. However, the poor agreement noted between nurses and panel of observers in the category of very urgent patients could be because of the possible difficulty in drawing the line between emergency very urgent and urgent categories. This could point to particular emphasis on criteria separating these categories whenever training is considered.

The present study was a record-based cross-sectional study conducted in one site, which was MDH-ED using existing data; it did not assess the proportion of self-trained nurses in the use of SATS or the years of experience in using SATS and how they performed. A prospective study that compares the performance of healthcare providers trained to those selves- trained for SATS may address the issue.

Nevertheless, findings of the study seemed to support limited-resource settings to use SATS even when formal training has not taken place. Thus, the findings were encouraging; we still recommend that institutions should make an effort in providing a formal training of SATS to obtain better results. The ED should make sure that resources are allocated according to the way patients are prioritised and provide services according to the level of priority.

### Study limitations

The healthcare providers were not formally trained on the use of SATS. However, the tool was still useful as it had an acceptable reliability and more than half of the patients were seen within the target waiting time from the triage to a consultation. Findings from our study cannot be generalised as it was conducted at one site. A prospective study can help to check the effect of formal training on the accuracy of classification in different priority level.

## Conclusion

The study aimed to determine the proportion of each priority level of patients, time of performance in each priority level and the reliability of the SATS tool at the MDH-ED. The profile of the priority-level categories at MDH-ED was made in the majority of routine and urgent patients. Almost all routine patients and three-quarters of emergency patients were seen for a consultation within the targeted time of 4 h and 10 min for the routine category and the emergency category, respectively.
