# Corrigendum: The effectiveness of the South African Triage Tool use in Mahalapye District Hospital – Emergency Department, Botswana

**DOI:** 10.4102/phcfm.v9i1.1695

**Published:** 2017-12-01

**Authors:** Stephane T. Tshitenge, Gboyega A. Ogunbanjo, Deogratias O. Mbuka

**Affiliations:** 1 Department of Family Medicine and Public Health, University of Botswana, Botswana, South Africa; 2 Department of Family Medicine and Primary Health Care, Sefako Makgatho Health Sciences University, South Africa

In the title of this article initially published, the spelling of ‘Traige Tool’ was unintentionally misprinted as ‘Triage Toll’. The authors sincerely regret this error. The title should be corrected as follows: The effectiveness of the South African Triage Tool use in Mahalapye District Hospital – Emergency Department, Botswana.

